# Vulvar Lobular Capillary Hemangioma: A Rare Location for a Frequent Entity

**DOI:** 10.1155/2016/3435270

**Published:** 2016-12-29

**Authors:** F. Abreu-dos-Santos, S. Câmara, F. Reis, T. Freitas, H. Gaspar, M. Cordeiro

**Affiliations:** ^1^Department of Gynecology and Obstetrics, Dr. Nélio Mendonça Hospital, Av. Luís de Camões, Funchal, 9004-514 Madeira Island, Portugal; ^2^Department of Pathologic Anatomy, Dr. Nélio Mendonça Hospital, Av. Luís de Camões, Funchal, 9004-514 Madeira Island, Portugal

## Abstract

Lobular capillary hemangioma, or pyogenic granuloma, is an acquired hemorrhagic benign vascular lesion of the skin and mucous membranes. The pyogenic granuloma of the vulva is a rare finding and a limited number of case reports are available in the literature. To the best of our knowledge this is the first case described as a single pyogenic granuloma on the vulva.

## 1. Introduction

Lobular capillary hemangioma or pyogenic granuloma, as it is usually known, is an acquired hemorrhagic benign vascular lesion of the skin and mucous membranes [1–3]. This hypervascularized lesion grows rapidly (in weeks or months) and usually presents as pedunculated or sessile mass, friable polypoid, and exophytic lesion, with a smooth or lobulated surface [1–4].

The pyogenic granuloma (PG) of the vulva is a rare finding and a limited number of case reports are available in the literature [1, 4–6].

## 2. Case Report

A 51-year-old woman, G3P3, was sent by her attending doctor to our hospital due to an abnormal lesion on the vulvar surface. The patient reported a lesion that initially looked like a small wart but continued growing for 10 months that in the end looked like a small cauliflower. She did not complain of pain but reported that the lesion was uncomfortable and sometimes bled.

**Figure 1:**
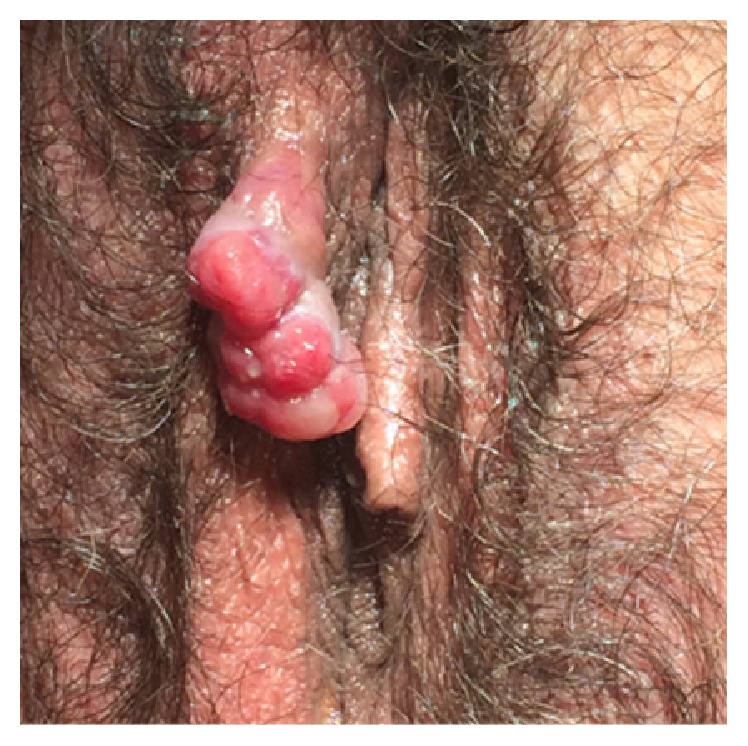
Image of the vulvar lobular capillary hemangioma.

On her medical history we notice hypertension and hyperthyroidism, controlled with medication. The gynecologic exam revealed a lobulated reddish malformation, ulcerated on the distal end which was located parallel to the clitoris, on the upper part of the right labia majora. The lesion was about 2 centimeters long (Figure 1). Her vaginal and cervical exams were normal.

**Figure 2:**
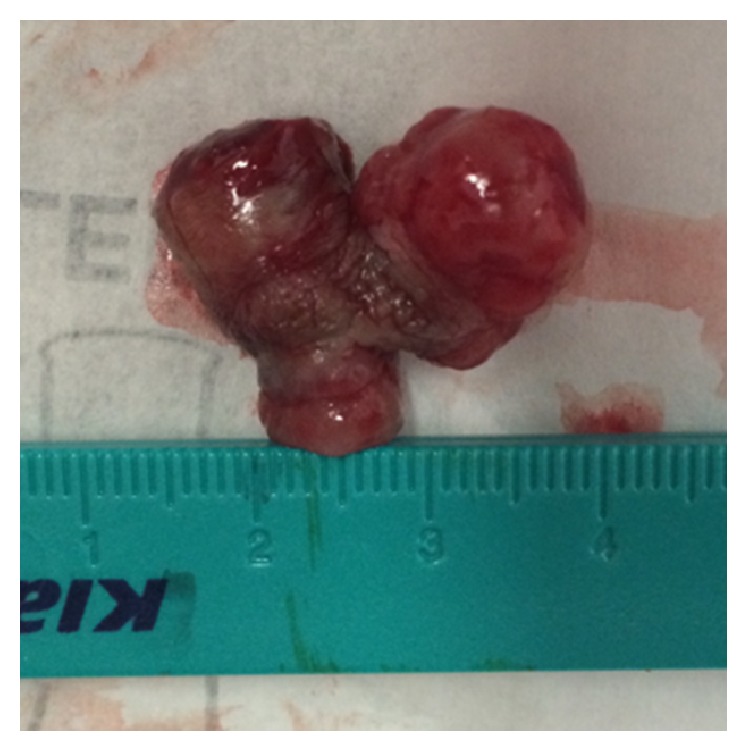
Vulvar lobular capillary hemangioma after excision: specimen sent to histopathological examination.

During the office visit we decided to do a wide excision under local anesthesia, and the whole lesion was removed (Figure 2). The specimen was then sent for histopathological examination.

**Figure 3:**
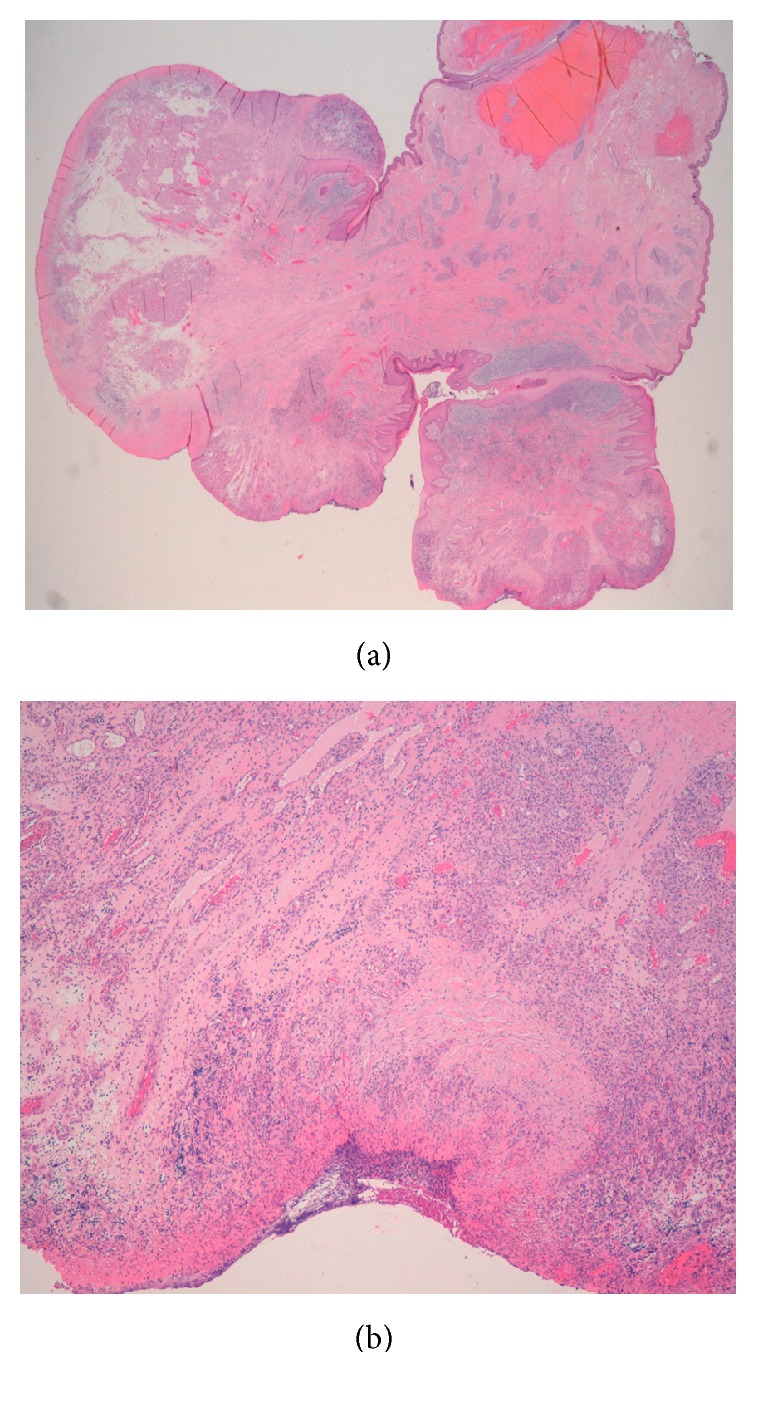
(a) View of the lesion with magnifying glass technique; (b) low power view (H&E 40x) showing the small vessels and ulceration of the lesion.

On the follow-up appointment, one month later, the scar was well healed and the patient had no complaints. The histopathological features ruled out vulvar neoplasia and were consistent with ulcerated lobular capillary hemangioma (Figure 3).

## 3. Discussion

The term “pyogenic granuloma” was first introduced by Hartzell in 1904, and only almost eighty years later was the histological term “lobular capillary hemangioma” introduced by Mills et al. [2]. The etiology of this disease is not yet fully understood. It has been considered to be a reactive hyperproliferative vascular response to a variety of stimuli, more than a true hemangioma [1, 3, 4]. Recently, the likely explanation for the pathogenesis is an excessive local production of tumor angiogenesis factor, as a result of minor trauma or an underlying cutaneous disease [4].

PG is a lesion of the skin and mucous membranes and it can be seen in various locations of the body. A 10-year retrospective analysis of 86 cases from Akamatsu et al. showed that the head and neck area, including the oral cavity and nasal mucosa, were the most commonly affected sites (56%, *n* = 46), followed by the upper limb (22%, *n* = 18), trunk (16%, *n* = 13), and lower limbs (6%, *n* = 5) [2]. On their report no vulvar cases were described.

The vulva is both a dermatologic and a gynecologic organ and, as such, may develop conditions more familiar to dermatologists than to gynecologists [7]. Unfamiliarity with these types of lesions, involving the vulva, can cause confusion with other polypoid lesions on this location [4, 7–10]. When faced with a lesion with such characteristics, it is necessary to consider a differential diagnosis that includes benign vulvar tumors, infectious lesions, skin cancers, and premalignant and malignant tumors [10].

There are various treatment options for PG: surgical excision, cryotherapy, sclerotherapy, curettage followed by electrocauterization, lasers, 5% imiquimod cream, and microembolization [2, 3]. Recently, there were reports of the use of a new topical treatment option, timolol, apparently with minimal adverse effects, easy administration, and good cosmetic outcomes [11, 12]. However, it may not be effective in all cases [11]. No vulvar cases of timolol use have been described.

Surgical excision and primary closure are associated with low recurrence rates among surgical treatments, being the most advised one. Besides that, it has some additional advantages, considering it is a single step treatment and the lesion can be sent to pathological evaluation [4]. Cryotherapy also has a low recurrence risk and, among the nonsurgical treatment options, is a good choice to sensitive areas like the face and neck [2, 3].

From the very few pyogenic granulomas of the vulva reported in the literature we only found cases of multiple PGs. So, to the best of our knowledge, ours is the first case described as a single pyogenic granuloma on the vulva.
